# *In vitro* assessment of selected Korean plants for antioxidant and antiacetylcholinesterase activities

**DOI:** 10.1080/13880209.2017.1397179

**Published:** 2017-11-08

**Authors:** Seulah Lee, Dahae Lee, Jiwon Baek, Eun Bee Jung, Ji Yun Baek, Il Kyun Lee, Tae Su Jang, Ki Sung Kang, Ki Hyun Kim

**Affiliations:** a School of Pharmacy, Sungkyunkwan University, Suwon, Republic of Korea;; b College of Korean Medicine, Gachon University, Seongnam, Republic of Korea;; c Research Center, Natural Medicine Research Team, Richwood Trading Company, LTD, Seoul, Republic of Korea;; d Institute of Green Bio Science & Technology, Seoul National University, Pyeong Chang, Korea

**Keywords:** *Prunella vulgaris* var. lilacina; *Oenothera biennis*; *Pharbitis nil*

## Abstract

**Context:** Antiacetylcholinesterase (AChE) drugs have been a main therapeutic treatment for Alzheimer’s disease because increased AChE levels play a key role in reducing neurotransmission.

**Objectives:** Extracts from 35 Korean plants were selected and screened for antioxidant and anti-cholinesterase activity to explore new sources derived from Korean natural resources that could be used as AD therapeutic agents.

**Materials and methods:** The antioxidant effect of extracts from 35 selected Korean plants was determined using two most common free radical scavenging assays using 1,1-diphenyl-2-picrylhydrazyl (DPPH) and 2,2′-azino-bis-3-ethylbenzthiazoline-6-sulphonic acid (ABTS). Additionally, the effect of extracts, identified as antioxidants, on acetylcholinesterase inhibition was assessed by an acetylcholinesterase assay kit.

**Results:** Out of 36 extracts of 35 plants tested, *Oenothera biennis* L. (9.09 μg/mL), *Saururus chinensis* (Lour.) Baill. (9.52 μg/mL) and *Betula platyphylla* var. *japonica* (9.85 μg/mL) showed strong DPPH scavenging activity. Twelve other extracts also exerted moderate free radical scavenging activities with IC_50_ values ranging from 10 to 50 μg/mL. Antioxidant capacity detected by ABTS assay was only significant in *O. biennis* (23.40 μg/mL), while the other extracts were weak or unable to reduce the production of ABTS. Based on the antioxidant activities of these plant extracts, 19 extracts with IC_50_ values less than 100 μg/mL in DPPH assay were selected for further AChE inhibition assay. Among the extracts tested, the IC_50_ value for *Prunella vulgaris* var. *lilacina* NAKAI (18.83 μg/mL) in AChE inhibitory activity was the lowest, followed by *O. biennis* (20.09 μg/mL) and *Pharbitis nil* Chosy (22.79 μg/mL).

**Conclusions:** Considering complex multifactorial etiology of AD, the extracts of *P. vulgaris* var. *lilacina* (aerial part), *O. biennis* (seed) and *P. nil* (seed) may be safe and ideal candidates for future AD modifying therapies.

## Introduction

Alzheimer’s disease (AD), a progressive age-related disease of the central nervous system (CNS), is characterized by deterioration in neurological function (Bartzokis 2004; Adewusi et al. 2011). AD is the most common type of dementia; 50–60% of dementia cases in the aging population are reported to be AD (Nordin et al. 1995). Although its exact cause remains uncertain, previous research has shown that lack of cholinergic neurotransmission and deposition of misfolded extracellular β-amyloid (Aβ) plaques and neurofibrillary tangles in the CNS are hallmarks of this disease (Ali et al. 2015). As the main pathological feature of AD, Aβ deposition prevents neurons from acquiring sufficient nutrition and causes increased levels of reactive oxygen metabolites (Ali et al. 2015; Haque and Nazir 2016; Wu et al. 2017).

Oxidative stress is known to be a key factor in the aging process. Since this is associated with Aβ plaque deposition which causes neuronal oxidative stress in AD patients, it is considered as a main pathogenesis cause of AD (Adewusi et al. 2011; Zhao et al. 2013). It was previously reported that reactive oxygen species (ROS) play an important role in neurodegenerative diseases (Zhao et al. 2013), and eventually contribute to neuronal death, ultimately causing impaired memory, cognitive ability and behavioral problems (Ali et al. 2015; Haque and Nazir 2016; Wu et al. 2017).

Recent studies have also elucidated the involvement of acetylcholinesterase (AChE) in AD cognitive deficits (Haque and Nazir 2016). The enzyme AChE hydrolyzes and breaks down acetylcholine (ACh) in the synaptic cleft. ACh is the neurotransmitter responsible for cholinergic transmission in the brain, and deposited within neurofibrillary tangles and Aβ plaques in the CNS (Dhanasekaran et al. 2015). The resulting lack of cholinergic neurotransmission due to reduced ACh levels eventually leads to cognitive deficits and in the worst cases, death (Adewusi et al. 2011; Ali et al. 2015). Thus, therapies that inhibit AChE and thereby increase ACh levels are promising temporary treatments for AD (Sallam et al. 2016).

AD’s multifactorial nature suggests that a multitargeted therapeutic approach might be more advantageous than single-target drugs and combination therapies. This has led to sustained searches by many research groups for natural drug candidates with antiamyloidogenic and antioxidant properties in addition to cholinesterase inhibitory activity (Mathew and Subramanian 2014). Natural products have been proven as antioxidant sources as well as AChE inhibitors (Adewusi et al. 2011; Mathew and Subramanian, 2014). The discovery of the naturally derived AChE inhibitors galantamine and rivastigmine, which are approved by the US Food and Drug Administration (FDA), has encouraged the search for other novel AD therapeutic agents from natural products (Mehta et al. 2012; Wang et al. 2016). Even though these AChE inhibitors are unable to prevent disease progression, they can improve cognitive dysfunction in mild to moderate AD cases (Wilcock et al. 2000; Schulz 2003). However, these inhibitors, along with other currently available inhibitors, are reported to have adverse side effects including gastrointestinal disturbances (Schulz 2003; Mukherjee et al. 2007; Adewusi et al. 2011). Thus, there is a need to discover new potent anti-AD agents with minimal side effects (Ali et al. 2015).

In this study, extracts from various plants originating in Korea were screened for antioxidant and AChE inhibitory activity. In the search for new anti-AD agents, these plants are appropriate to screen since the plants themselves and health supplement formulations derived from them are well tolerated with few side effects. They also possess definite beneficial effects such as anti-inflammatory and antioxidant activity, which are related to brain function. In this study, 35 different plants traditionally used in Korea for rejuvenation, anti-inflammation and/or improving memory and cognitive function were selected (Table 1) (Zee 2009). The current study is the first attempt to identify and compare potential antioxidant and AChE inhibition candidates from these plants.

**Table 1.** Details of the Korean plants used in the current study and their usage related to effects on the CNS/cognitive functions.

Botanical name	Family	Common name	Usage (Zee 2009)	Sampling location (Collection time)
*A. bidentata* Blume	Amaranthaceae	Ox knee	Anti-inflammation	Suwon, Korea (July 2013)
*A. japonica* (Miq.) Nakai	Amaranthaceae	Chaff flower	Antioxidant	Suwon, Korea (July 2013)
*A. rugosa*	Lamiaceae	Korean mint	Antioxidant	National Institute of Horticultural and Herbal Science (NIHHS) (May 2013)
*A. tuberosum* Roth.	Liliaceae	Garlic chives	Antioxidant	Suwon, Korea (July 2012)
*A. continentalis* KITAGAWA	Araliaceae	Dok Hwal	Neuroprotection Antioxidant	NIHHS (May 2013)
*A. capillaris* Thunb.	Compositae	Redstem wormwood	Anti-inflammation	NIHHS (May 2013)
*A. koraiensis* NAKAI	Compositae	Korean starwort	Antioxidant	Suwon, Korea (July 2013)
*A. tartaricus* L.	Compositae	Aster	Antioxidant	Suwon, Korea (July 2013)
*A. japonica* Koidz.	Compositae	Atractylodes	Anti-inflammation	Suwon, Korea (July 2012)
*B. platyphylla* var. *japonica*	Betulaceae	White birch	Anti-inflammation	Chungcheongbuk-do, Korea (May 2012)
*B. falcatum* L.	Apiaceae	Sickle hare’s ear	Anti-inflammation	Jeollanam-do, Korea (June 2012)
*B. koreana* Nakai ex Chung & al.	Buxaceae	Korean box tree	Against neuralgia, rheumatism,	NIHHS (May 2013)
*C. setidens* NAKAI	Compositae	Korean gondre thistle	Anti-inflammation	Suwon, Korea (July 2012)
*C. pilosula* (FR.) NANNF	Campanulaceae	Pilose asiabell	Promotes growth of neurons	Suwon, Korea (July 2012)
*E. ciliata* (Thunb.) Hyl.	Lamiaceae	Vietnamese balm	Anti-inflammation	Suwon, Korea (July 2013)
*F. japonica* (Houtt.) Ronse Decr.	Polygonaceae	Knotweed	Sedative	NIHHS (May 2013)
*F. vulgare* Mill.	Umbelliferae (Apiaceae)	Fennel	Antioxidant	NIHHS (May 2013)
*G. scabra* Bunge for. Scabra	Gentianaceae	Korean gentian	Anti-inflammation	Suwon, Korea (July 2013)
*H. dulcis* Thunb.	Rhamnaceae	Raisin tree	Anti-inflammation	Gangwon-do, Korea (May 2014)
*I. tinctoria* var. *yezoensis* OHWI	Cruciferae	Woad	Antioxidant	Suwon, Korea (July 2013)
*L. indica* var. *laciniata*	Compositae	Indian lettuce	Sedative	Suwon, Korea (July 2012)
*L. japonicus* Houtt.	Lamiaceae	Chinese motherwort	Antioxidant	NIHHS (May 2013)
*L. sibiricus* Linne	Labiatae	Honeyweed	Antioxidant	Suwon, Korea (July 2013)
*M. charantia* L.	Cucurbitaceae	Bitter gourd	Antidiabetic	Suwon, Korea (July 2013)
*O. biennis* L.	Onagraceae	Evening primrose	Anti-inflammation	NIHHS (May 2013)
*O. japonicus* KER-GAWLER	Liliaceae	Liriope tuber	Protects brain cells, promotes growth of neurons	Suwon, Korea (July 2012)
*P. nil* Chosy	Convolvulaceae	Morning glory	Anti-inflammation	Suwon, Korea (July 2012)
*P. amurense* Rupr.	Rutaceae	Amur cork tree	Improves memory	Suwon, Korea (July 2012)
*P. vulgaris* var. *lilacina* NAKAI	Labiatae	Common self-heal	Antiaging	Suwon, Korea (July 2013)
*R. glutinosa* (GAERTNER) LIBOSCHITZ	Scrophulariaceae	Rehmannia root	Protection of brain cells, improves memory	Suwon, Korea (July 2013)
*S. chinensis* (Lour.) Baill.	Saururaceae	Lizard’s tail	Protection of brain cells	Suwon, Korea (July 2013)
*S. tenuifolia* (Benth.) Briq.	Lamiaceae	Schizonepeta spike	Antiaging	Suwon, Korea (July 2012)
*S. buergeriana* Miq.	Scrophulariaceae	Scrophularia root	Anti-inflammation	Suwon, Korea (July 2013)
*Senna tora* (L.) Roxb.	Leguminosae	Cassia seed	Antiaging	NIHHS (May 2013)
*T. kirilowii* Maxim	Cucurbitaceae	Chinese cucumber	Anti-inflammation	Suwon, Korea (July 2013)

## Materials and methods

### Chemicals

1,1-Diphenyl-2-picrylhydrazyl (DPPH), 2,2′-azino-bis-3-ethylbenzthiazoline-6-sulphonic acid (ABTS), ascorbic acid (vitamin C) and donepezil hydrochloride were purchased from Sigma Aldrich (Seoul, Korea). The other chemicals and reagents used were of high quality and obtained from commercial sources.

### Plant materials

All plant materials were procured from the National Institute of Horticultural and Herbal Science (NIHHS), Eumseong-gun, Chungcheongbuk-do, Korea, and authenticated by Dr Seung-Eun Lee, Department of Herbal Crop Research, NIHHS, Eumseong-gun, Chungcheongbuk-do, Korea. Voucher specimens of the materials [*A bidentata* (AB-2013-07), *A. japonica* (AJ-2013-07), *Agastache rugose* (AR-2013-05), *Allium tuberosum* (AT-2012-07), *Aralia continentalis* (AC-2013-05a), *Artemisia capillaris* (AC-2013-05 b), *Aster koraiensis* (AK-2013-07), *A. tartaricus* (AT-2013-07), *Atractylodes japonia* (AJ-2012-07), *Betula platyphylla* var. *japonica* (BPJ-2012-05), *Bupleurum falcatum* (BF-2012-06), *Buxus koreana* (BK-2013-05), *Cirsium setidens* (CS-2012-07), *Codonopsis pilosula* (CP-2012-07), *Elsholtzia ciliata* (EC-2013-07), *Fallopia japonica* (FJ-2013-05), *Foeniculum vulgare* (FV-2013-05), *Gentiana scabra* (GS-2013-07), *Hovenia dulcis* (HD-2014-05), *Isatis tinctoria* var. *yezoensis* (ITY-2013-07), *Lactuca indica* var. *laciniata* (LIL-2012-07), *Leonurus japonicus* (LJ-2013-05), *L. sibiricus* (LS-2013-07), *Momordica charantia* (MC-2013-07), *Oenothera biennis* (OB-2013-05), *Ophiopogon japonicus* (OJ-2012-07), *Pharbitis nil* (PN-2012-07), *Phellaodendron amurense* (PA-2012-07), *Prunella vulgaris* var. *lilacina* (PVL-2013-07), *Rehmannia glutinosa* (RG-2013-07), *Saururus chinensis* (SC-2013-07), *Schizonepeta tenuifolia* (ST-2012-07), *Scrophularia buergeriana* (SB-2013-07), *Senna tora* (ST-2013-05) *Trichosanthes kirilowii* (TK-2013-07)] were deposited at the Herbarium Conservation Center of the NIHHS. No permits were required for these procurements. The botanical name, family, common name, sampling location and usage related to CNS effect/cognitive functions are summarized in Table 1.

### Plant extract preparation

Freshly collected plant materials were dried in a hot air oven at 55 °C and then pulverized. Plant parts used are presented in Table 2. To prepare the extracts, 5 g of each powdered plant was extracted using the indicated solvent under different conditions (Table 2) and each filtered extract was concentrated by complete evaporation in a vacuum centrifuge. The dried extract was then stored at −20 °C. The entire study was conducted using a single batch of each plant extract to avoid batch-to-batch variation and maximize product consistency.

**Table 2.** Antioxidant activities of 36 different extracts from 35 selected plants.

Test samples	Part	Extract solvent	IC_50_ (μg/mL)^a^	IC_50_ (μg/mL)^a^
			DPPH	ABTS
*A. bidentata*	RT	100% MeOH	>200	>200
*A. japonica*	RT	100% MeOH	>200	>200
*A. rugosa*	ST	100% H_2_O	>200	>200
*A. tuberosum*	AP	100% MeOH	169.32	>200
*A. continentalis*	ST	100% H_2_O	>200	>200
*A. capillaris*	AP	100% EtOH	13.17	114.87
*A. koraiensis*	AP	100% MeOH	44.23	94.68
*A. tartaricus*	FL	100% EtOH (74 °C)	11.28	>200
*A. japonia*	BU	100% MeOH	194.54	>200
*B. platyphylla* var. *japonica*	BA	80% EtOH/H_2_O	9.85	>200
*B. falcatum*	RT	100% EtOH (74 °C)	>200	>200
*B. koreana*	ST	100% H_2_O	107.79	>200
*C. setidens*	AP	100% MeOH	36.20	>200
*C. pilosula*	AP	100% EtOH (85 °C)	32.44	>200
*E. ciliata*	AP	100% MeOH	69.26	183.08
*F. japonica*	ST	100% EtOH	14.03	96.41
*F. vulgare*	ST	100% EtOH	123.02	>200
*G. scabra*	RT	100% MeOH	>200	>200
*H. dulcis*	TW	100% MeOH	29.17	189.62
*I. tinctoria* var. *yezoensis*	AP	100% EtOH (85 °C)	32.14	133.46
*L. indica* var. *laciniata*	RT	100% MeOH	>200	>200
*L. japonicus*	ST	100% EtOH	52.77	>200
*L. sibiricus*	AP	100% MeOH	83.85	137.63
*M. charantia*	AP	100% MeOH	>200	>200
*O. biennis*	SE	100% MeOH	9.09	23.40
*O. japonicus*	WP	100% MeOH	131.80	>200
*P. nil*	SE	100% MeOH	33.75	>200
*P. amurense*	BA	100% EtOH (74 °C)	124.58	>200
*P. vulgaris* var. *lilacina*	AP	100% EtOH (85 °C)	18.13	>200
*R. glutinosa*	RT	100% EtOH (74 °C)	>200	>200
*S. chinensis*	AP	100% MeOH	9.52	>200
*S. tenuifolia*	AP	100% MeOH	43.87	>200
*S. buergeriana*.	AP	100% MeOH	>200	>200
*S. buergeriana*	RT	100% MeOH	48.10	>200
*Senna tora*	SE	100% H_2_O	>200	>200
*T. kirilowii*	AP	100% EtOH (74 °C)	61.19	79.39
Ascorbic acid^b^			4.97	9.88

a IC_50_ values were determined by curve-fitting the data points using nonlinear regression.

b Ascorbic acid was used as a positive control for DPPH and ABTS radical scavenging activities.

WP: whole plant; RT: root; FL: flower; AP: aerial part; ST: stem; SE: seed; BU: bulb; BA: bark; TW: twig.

### Determination of antioxidant activity by scavenging effect on 2,2’-diphenyl-1-picryl hydrazyl radical (DPPH)

In each well of 96-well microplate, 100 μL aqueous solution from the sample (control: 100 μL of distilled water) was added to an ethanolic solution of DPPH (100 μL, 60 μM) based on a previously reported method with minor modifications (Eom et al. 2016). The absorbance at 540 nm was measured using a microplate reader (Tecan SPECTRAFluor; Tecan UK, Goring-on-Thames, UK) after mixed gently and allowed to stand at room temperature for 30 min. Ascorbic acid was used as a DPPH scavenging positive control.

### Determination of antioxidant activity by scavenging effect on 2,2′-azino-bis-3-ethylbenzthiazoline-6-sulphonic acid (ABTS)

The ABTS activity was measured according to the previously reported method (Moreno-Montoro et al. 2017). After adding 0.5 mL of sample to 3 mL of the diluted ABTS solution, the absorbance was measured at 415 nm using a spectrophotometer. The ABTS radical scavenging (%) was calculated as (1−*A*/*A*_0_) × 100, where *A*_0_ is the absorbance of the control, and *A* is the absorbance of the samples. The IC_50_ value was calculated as the concentration of sample required to scavenge 50% of free radicals. Ascorbic acid was used as an ABTS scavenging positive control.

### AChE enzyme activity assay

Acetylcholinesterase inhibition activity was determined using an acetylcholinesterase assay kit according to the manufacturer’s protocol (BioVision, Milpitas, CA). In brief, 50 µL sample and assay buffer was added to each well of a 96-well plate, followed by the addition of 50 µL of the reaction mixture. After incubation at 37 °C for 20 min, the absorbance at 570 nm was measured using a microplate reader (PowerWave XS; Bio-Tek Instruments, Winooski, VT).

### Statistical analysis

Statistical significance was determined with analysis of variance (ANOVA) followed by a multiple comparison test with Bonferroni’s adjustment. *p* Values less than 0.05 were considered statistically significant. Analyses were performed using SPSS ver. 19.0 (SPSS Inc., Chicago, IL).

## Results

### Free radical scavenging activity of the extracts using DPPH and ABTS assay

Multiple factors are involved in the development of neurodegenerative disease. Considering AD’s complex multifactorial etiology, phytochemicals that have antioxidant as well as AChE inhibitory activity have been considered to be safer and better therapeutic candidates for treating AD (Parodi et al. 2015). Antioxidant therapy has proven successful for improving cognitive function and behavioral deficits in patients with mild to moderate AD (Gutzmann and Hadler 1998).

The antioxidant activity of extracts from 35 selected Korean plants (Table 1) was determined using the free radical DPPH. IC_50_ values for DPPH radical scavenging were determined based on the concentration of the extract required for approximately 50% of the original activity. IC_50_ values for all extracts are presented in Table 2. Strong IC_50_ values were obtained for *O. biennis* L. (9.09 μg/mL), *S. chinensis* (Lour.) Baill. (9.52 μg/mL) and *B. platyphylla* var. *japonica* (9.85 μg/mL), whereas the value for ascorbic acid was 4.97 μg/mL. *F. japonica* (Houtt.) Ronse Decr., *A. capillaris* Thunb., *S. buergeriana* Miq., *S. tenuifolia* (Benth.) Briq., *A. koraiensis* NAKAI, *C. pilosula* (FR.) NANNF, *I. tinctoria* var. *yezoensis* OHWI, *A. tartaricus* L., *H. dulcis* Thunb, *P. vulgaris* var. *lilacina* NAKAI, *P. nil* Chosy and *C. setidens* NAKAI also exerted moderate free radical scavenging activities with IC_50_ values ranging from 10 to 50 μg/mL (Table 2). Interestingly, the MeOH extract from the roots of *S. buergeriana* Miq. exhibited good DPPH scavenging activity with a corresponding IC_50_ value of 48.10 μg/mL, whereas the MeOH extract from its aerial parts was not active (IC_50_ > 200 μg/mL). In addition, the extracts of 35 selected Korean plants were investigated for their antioxidant properties using ABTS radical scavenging capacity assay (Table 2). Antioxidant capacity detected by ABTS assay was only significant in *O. biennis* (23.40 μg/mL), while the other extracts were weak or unable to reduce the production of ABTS (Table 2).

### Acetylcholinesterase (AChE) inhibitory activity of the selected extracts

Based on plant extract antioxidant activities, 19 extracts with IC_50_ values less than 100 μg/mL in DPPH assay were selected for further AChE inhibition assays. The main pathological feature that characterizes AD is reduction in cholinergic acetylcholine (ACh) neurotransmission (Bae and Lee 2015; Li et al. 2016; Xu et al. 2016). Acetylcholinesterase (AChE) is responsible for ACh hydrolysis. Reduced levels of ACh eventually lead to cognitive dysfunction and even death. Thus, AChE inhibition is considered as the most valuable therapy for AD (Sim et al. 2014). Although this disease cannot be prevented from progressing, there are various AChE inhibitors currently available that can improve symptoms in mild to moderate AD patients (Schulz 2003; Mehta et al. 2012). However, intensive research is still needed to discover new candidates against AD since the current AChE inhibitors carry adverse effects (Wilcock et al. 2000; Mehta et al. 2012). 19 extracts selected based on their antioxidant activity were tested for AChE inhibitory activity and the results are shown in Table 3 represent % inhibition at 100 mg/mL and the IC_50_ for tested extracts. Donepezil hydrochloride was used as the standard AChE inhibitor in this study which showed an IC_50_ of 0.03 μg/mL. The IC_50_ value for AChE inhibitory activity was the lowest for *P. vulgaris* var. *lilacina* (18.83 μg/mL) followed by *O. biennis* (20.09 μg/mL) and *P. nil* (22.79 μg/mL). These plant extracts also showed very high antioxidant activity in the DPPH assay. In addition, *O. biennis* exhibited significant activity in reducing the production of ABTS (Table 2).

**Table 3.** AChE inhibition assays for 19 extracts of selected plants with antioxidant activity.

Test samples^a^	% AChE inhibition at 100 μg/mL	IC_50_ (μg/mL)^b^
*A. capillaris*	101.39 ± 0.66	>200
*A. koraiensis*	97.06 ± 3.28	>200
*A. tartaricus*	93.34 ± 0.66	>200
*B. platyphylla* var. *japonica*	89.63 ± 5.90	>200
*C. setidens*	91.79 ± 1.09	>200
*C. pilosula*	102.47 ± 4.81	>200
*E. ciliata*	68.27 ± 1.09	>200
*F. japonica*	64.09 ± 3.50	>200
*H. dulcis*	96.44 ± 1.09	>200
*I. tinctoria* var. *yezoensis*	87.00 ± 3.06	>200
*L. japonicus*	95.67 ± 2.63	>200
*L. sibiricus*	82.82 ± 1.97	>200
*O. biennis*	25.41 ± 0.43	20.09
*P. nil*	39.16 ± 1.31	22.79
*P. vulgaris* var. *lilacina*	39.03 ± 0.87	18.83
*S. chinensis*	59.15 ± 2.65	>200
*S. tenuifolia*	92.42 ± 2.41	>200
*S. buergeriana*	109.90 ± 1.31	>200
*T. kirilowii*	96.75 ± 0.66	>200
Donepezil hydrochloride^c^	92.42 ± 3.72	0.03

a Tested extracts were the same as those used for the antioxidant assay.

b IC_50_ values were determined by curve-fitting the data points using nonlinear regression.

c Donepezil hydrochloride was used as a positive control for the AChE inhibition activity.

## Discussion

Out of 35 Korean plants screened, the extracts of dry plant parts from *P. vulgaris* var. *lilacina* (aerial part), *O. biennis* (seed) and *P. nil* (seed) were selected as candidate sources for potent AChE inhibitors as well as antioxidants. Due to AD’s multifactorial pathogenesis, multi-targeted drugs are preferred as an effective therapeutic strategy. These selected Korean plants exhibiting *in vitro* AChE inhibition and antioxidant activity act on multiple therapeutic AD targets and can be consumed daily in our diet to provide their neuroprotective effects.

*Oenothera biennis* (Onagraceae) is commonly known as evening primrose and its seeds are known for their high antioxidant activities (Budinčević et al. 1995). The seeds of *O. biennis* are also believed to have medicinal value mainly due to the presence of *γ*-linolenic acid, which is known to be an essential dietary supplementation for humans. *γ*-Linolenic acid improves many pathological conditions including dermatitis, platelet aggregation and high blood pressure (Corrigan et al. 1998; Barre 2001). *γ*-Linolenic acid is an omega-6 (*n*-6) fatty acid, which is known to have anti-inflammatory activity along with omega-3 (*n*-3) fatty acids and affects the pathogenesis of many diseases where inflammation plays a critical role including cancer, diabetes, heart disease and AD (Kapoor and Huang 2006). In one study, *n*-6 fatty acids were reported to contribute to the improvement in learning tasks and recovery from neurotoxins, which suggested the potential for *O. biennis* seeds to be utilized as a candidate therapeutic agent for AD (Yehuda et al. 1996). The crude extract from *O. biennis* seeds was also reported to have antioxidant activities due to the involvement of phenolic constituents (Wettasinghe et al. 2002). In this work, the MeOH extract from *O. biennis* seeds showed AChE inhibitory effects with an IC_50_ value of 20.09 μg/mL, which was the highest antioxidant activity among the tested extracts.

The aerial parts of *P. vulgaris* var. *lilacina* (Labiatae), which have been used in Chinese folk medicine to calm irritated skin and heal wounds, are rich in phenolic acids such as rosmarinic acid, caffeic acid and kaempferol. Rosmarinic acid is the main component in the aerial parts of *P. vulgaris* var. *lilacina*. It has been shown to exhibit antioxidant effects by suppressing lipoperoxidation and scavenging superoxide radicals (Škottová et al. 2004). In addition, it also inhibits the formation of β-amyloid (Aβ) plaques. Deposition of misfolded extracellular Aβ plaques is one of the main possible causes of AD. The inhibitory activity of rosmarinic acid eventually protects against memory impairment (Alkam et al. 2007). Caffeic acid, one of the main components of the aerial parts of *P. vulgaris* var. *lilacina*, is well-known for its antioxidant and anti-inflammatory activities and was also found to possess a significant protective effect against β-amyloid-induced neurotoxicity by inhibiting calcium influx and tau phosphorylation (Sul et al. 2009). Kaempferol, another major component of *P. vulgaris* var. *lilacina*, has been found to protect PC12 and T47D cells from β-amyloid-induced toxicity (Roth et al. 1999). In this regard, the aerial parts of *P. vulgaris* var. *lilacina*, with these phytochemicals as the major content, should be further assessed as an adjuvant therapeutic agent for AD. In this study, the MeOH extract from the aerial parts of *P. vulgaris* var. *lilacina* was tested for AChE inhibition as well as antioxidant activity and it showed the highest AChE inhibition with an IC_50_ value of 18.83 μg/mL along with high antioxidant activity.

*Pharbitis nil* (Convolvulaceae) is known as morning glory and the seeds of *P. nil* (Pharbitidis Semen) have been used as a purgative drug in folkloric medicine in Asian countries (Kim et al. 2009). The seeds of *P. nil* are reported to be a rich source of diverse phytochemicals, including resin glycosides, gibberellins, flavonoids, anthocyanins, diterpenoids, lignans, triterpene saponins and phenolic compounds (Kim et al. 2008, 2009, 2010, 2011, 2013, 2014; Park et al. 2016). The bioactivity of the constituents of *P. nil* is still underexplored; however, polysaccharides from *P. nil* seeds were recently reported to possess antioxidant activities (Wang et al. 2014). Although study on the possible medicinal usage of *P. nil* seeds is still limited, the properties of phytochemicals such as polysaccharides, flavonoids, diterpenoids and phenolic compounds suggest the potential use of *P. nil* seeds as a treatment for AD. Flavonoids are known to possess neuroprotective properties that improve cognitive function, and show protective effects against memory deficits associated with normal aging (Macready et al. 2009). There has also been a report on the potential AChE inhibitory activity of diterpenoids, which provides a theoretical basis for further research and utilization of *P. nil* seeds for cholinesterase inhibitory activity (Hung et al. 2011). In addition, some of the diterpenes, lignans and phenolic compounds isolated from *P. nil* seeds were found to show anti-neuroinflammatory activity by inhibiting nitric oxide (NO) production in lipopolysaccharide (LPS)-activated BV-2 microglia cells (Kim et al. 2011, 2013, 2014). Under pathological conditions, microglia cells, which are the immune resident cells of the brain, are over-activated and produce a variety of pro-inflammatory mediators including NO, which consequently leads to various neurodegenerative conditions of the CNS including Parkinson’s and Alzheimer’s disease (Kim et al. 2015; Suh et al. 2016). In the current study, the MeOH extract from the seeds of *P. nil* was tested for antioxidant activity and showed high activity in scavenging reactive oxygen species, which led to a subsequent assay to observe its AChE inhibitory effects. The extract showed AChE inhibition with an IC_50_ value of 22.79 μg/mL.

## Conclusions

The extracts from plants originating in Korea, which have been used in Korea for rejuvenation and anti-inflammation and/or improving memory and cognitive function, were screened for AChE inhibition and antioxidant activity for the first time. Of the 35 plant materials tested, the extracts from the aerial part of *P. vulgaris* var. *lilacina*, the seeds of *O. biennis* and the seeds of *P. nil* were selected as promising candidates for sources of potent AChE inhibitors as well as antioxidants. Considering the complex multifactorial etiology of AD, these selected plant extracts may be safe and ideal candidates as therapies against AD. Further evaluation to identify active ingredients and assess safety and bioavailability using *in vivo* animal models is required.

## References

[CIT0001] Adewusi EA, Moodley N, Steenkamp V. 2011 Antioxidant and acetylcholinesterase inhibitory activity of selected southern African medicinal plants. S Afr J Bot. 77:638–644.

[CIT0002] Ali MY, Jung HA, Choi JS. 2015 Anti-diabetic and anti-Alzheimer’s disease activities of Angelica decursiva . Arch Pharm Res. 38:2216–2227. 26152875 10.1007/s12272-015-0629-0

[CIT0003] Alkam T, Nitta A, Mizoguchi H, Itoh A, Nabeshima T. 2007 A natural scavenger of peroxynitrites, rosmarinic acid, protects against impairment of memory induced by Aβ_25-35_. Behav Brain Res. 180:139–145. 17420060 10.1016/j.bbr.2007.03.001

[CIT0004] Bae JR, Lee BD. 2015 Function and dysfunction of leucine-rich repeat kinase 2 (LRRK2): Parkinson’s disease and beyond. BMB Rep. 48:243–248. 25703537 10.5483/BMBRep.2015.48.5.032 PMC4578562

[CIT0005] Barre DE. 2001 Potential of evening primrose, borage, black currant, and fungal oils in human health. Ann Nutr Metab. 45:47–57. 11359029 10.1159/000046706

[CIT0006] Bartzokis G. 2004 Age-related myelin breakdown: a developmental model of cognitive decline and Alzheimer’s disease. Neurobiol Aging. 25:5–18. 14675724 10.1016/j.neurobiolaging.2003.03.001

[CIT0007] Budinčević M, Vrbaški Ž, Turkulovxyc J, Dimić E. 1995 Antioxidant activity of *Oenothera biennis* L. Fett Wiss Technol. Eur J Lipid Sci Tech. 97:277–280.

[CIT0008] Corrigan FM, Horrobin DF, Skinner ER, Besson JAO, Cooper M. 1998 Abnormal content of *n*-6 and *n*-3 long-chain unsaturated fatty acids in the phosphoglycerides and cholesterol esters of parahippocampal cortex from Alzheimer’s disease patients and its relationship to acetyl CoA content. Int J Biochem Cell Biol. 30:197–207. 9608673 10.1016/s1357-2725(97)00125-8

[CIT0009] Dhanasekaran S, Perumal P, Palayan M. 2015 *In-vitro* screening for acetylcholinesterase enzyme inhibition potential and antioxidant activity of extracts of *Ipomoea aquatic* Forsk: therapeutic lead for Alzheimer’s disease. J App Pharm Sci. 5:12–16.

[CIT0010] Eom HJ, Kang HR, Kim HK, Jung EB, Park HB, Kang KS, Kim KH. 2016 Bioactivity-guided isolation of antioxidant triterpenoids from *Betula platyphylla* var. *japonica* bark. Bioorg Chem. 66:97–101. 27060627 10.1016/j.bioorg.2016.04.001

[CIT0011] Gutzmann H, Hadler D. 1998 Sustained efficacy and safety of idebenone in the treatment of Alzheimer’s disease: update on a 2-year double-blind multicentre study. J Neural Transm Suppl. 54:301–310. 9850939 10.1007/978-3-7091-7508-8_30

[CIT0012] Haque R, Nazir A. 2016 Identification and functional characterization of a putative IDE, C28F5.4 (ceIDE-1), in *Caenorhabditis elegans*: implications for Alzheimer’s disease. Biochim Biophys Acta. 1860:2454–2462. 27443962 10.1016/j.bbagen.2016.07.013

[CIT0013] Hung TM, Luan TC, Vinh BT, Cuong TD, Min BS. 2011 Labdane-type diterpenoids from *Leonurus heterophyllus* and their cholinesterase inhibitory activity. Phytother Res. 25:611–614. 20981867 10.1002/ptr.3307

[CIT0014] Kapoor R, Huang Y-S. 2006 Gamma linolenic acid: an antiinflammatory omega-6 fatty acid. Curr Pharm Biotechnol. 7:531–534. 17168669 10.2174/138920106779116874

[CIT0019] Kim KH, Choi SU, Son MW, Choi SZ, Clardy J, Lee KR. 2013 Pharbinilic acid, an allogibberic acid from morning glory (*Pharbitis nil*). J Nat Prod. 76:1376–1379. 23815260 10.1021/np400326e

[CIT0016] Kim KH, Choi SU, Lee KR. 2009 Diterpene glycosides from the seeds of *Pharbitis nil*. J Nat Prod. 7:1121–1127. 10.1021/np900101t 19435339

[CIT0017] Kim KH, Choi SU, Son MW, Lee KR. 2010 Two new phenolic amides from the seeds of *Pharbitis nil*. Chem Pharm Bull. 58:1532–1535. 21048350 10.1248/cpb.58.1532

[CIT0018] Kim KH, Ha SK, Choi SU, Kim SY, Lee KR. 2011 Bioactive phenolic constituents from the seeds of *Pharbitis nil*. Chem Pharm Bull. 59:1425–1429. 22041085 10.1248/cpb.59.1425

[CIT0015] Kim KH, Jin MR, Choi SU, Son MW, Lee KR. 2008 Three new *ent*-kaurane diterpenoids from the seeds of *Pharbitis nil*. Heterocycles. 75:1447–1455.

[CIT0021] Kim KH, Moon E, Lee SR, Park KJ, Kim SY, Choi SU, Lee KR. 2015 Chemical constituents of the seeds of *Raphanus sativus* and their biological activity. J Braz Chem Soc. 26:2307–2312.

[CIT0020] Kim KH, Woo KW, Moon E, Choi SU, Kim SY, Choi SZ, Son MW, Lee KR. 2014 Identification of antitumor lignans from the seeds of morning glory (*Pharbitis nil*). J Agric Food Chem. 62:7746–7752. 25020073 10.1021/jf501470k

[CIT0022] Li N, Liu Y, Li W, Zhou L, Li Q, Wang X, He P. 2016 A UPLC/MS-based metabolomics investigation of the protective effect of ginsenosides Rg1 and Rg2 in mice with Alzheimer’s disease. J Ginseng Res. 40:9–17. 26843817 10.1016/j.jgr.2015.04.006 PMC4703800

[CIT0023] Macready AL, Kennedy OB, Ellis JA, Williams CM, Spencer JPE, Butler LT. 2009 Flavonoids and cognitive function: a review of human randomized controlled trial studies and recommendations for future studies. Genes Nutr. 4:227–242. 19680703 10.1007/s12263-009-0135-4 PMC2775887

[CIT0024] Mathew M, Subramanian S. 2014 *In vitro* screening for anti-cholinesterase and antioxidant activity of methanolic extracts of Ayurvedic medicinal plants used for cognitive disorders. PLoS One. 9:e86804. 24466247 10.1371/journal.pone.0086804 PMC3900633

[CIT0025] Mehta M, Adem A, Sabbagh M. 2012 New acetylcholinesterase inhibitors for Alzheimer’s disease. Int J Alzheimers Dis. 2012:728983 22216416 10.1155/2012/728983 PMC3246720

[CIT0026] Moreno-Montoro M, Olalla-Herrera M, Rufián-Henares JÁ, Martínez RG, Miralles B, Bergillos T, Navarro-Alarcón M, Jauregi P. 2017 Antioxidant, ACE-inhibitory and antimicrobial activity of fermented goat milk: activity and physicochemical property relationship of the peptide components. Food Funct. 8:2783–2791. 28702643 10.1039/c7fo00666g

[CIT0027] Mukherjee PK, Kumar V, Mal M, Houghton PJ. 2007 Acetylcholinesterase inhibitors from plants. Phytomedicine. 14:289–300. 17346955 10.1016/j.phymed.2007.02.002

[CIT0028] Nordin S, Monsch AU, Murphy C. 1995 Unawareness of smell loss in normal aging and Alzheimer’s disease: discrepancy between self-reported and diagnosed smell sensitivity. J Gerontol Psychol Sci. 50B:187–192. 10.1093/geronb/50b.4.p187 7606530

[CIT0029] Park YJ, Choi CI, Chung KH, Kim KH. 2016 Pharbilignan C induces apoptosis through a mitochondria-mediated intrinsic pathway in human breast cancer cells. Bioorg Med Chem Lett. 26:4645–4649. 27575473 10.1016/j.bmcl.2016.08.054

[CIT0030] Parodi J, Ormeño D, Ochoa-de la Paz LD. 2015 Amyloid pore-channel hypothesis: effect of ethanol on aggregation state using frog oocytes for an Alzheimer’s disease study. BMB Rep. 48:13–18. 25047445 10.5483/BMBRep.2015.48.1.125 PMC4345636

[CIT0031] Roth A, Schaffner W, Hertel C. 1999 Phytoestrogen kaempferol (3,4’,5,7-tetrahydroxyflavone) protects PC12 and T47D cells from beta-amyloid-induced toxicity . J Neurosci Res. 57:399–404. 10412031

[CIT0032] Sallam A, Mira A, Ashour A, Shimizu K. 2016 Acetylcholine esterase inhibitors and melanin synthesis inhibitors from *Salvia officinalis*. Phytomedicine. 23:1005–1011. 27444345 10.1016/j.phymed.2016.06.014

[CIT0033] Schulz V. 2003 Ginkgo extract or cholinesterase inhibitors in patients with dementia: what clinical trials and guidelines fail to consider. Phytomedicine. 10:74–79. 12807348 10.1078/1433-187x-00302

[CIT0034] Sim JY, Kim MA, Kim MJ, Chun WJ, Kwon YS. 2014 Acetylcholinesterase inhibitors from the stem of *Zea mays*. Nat Prod Sci. 20:13–16.

[CIT0035] Škottová N, Kazdová L, Oliyarnyk O, Vecera R, Sobolová L, Ulrichová J. 2004 Phenolics-rich extracts from *Silybum marianum* and *Prunella vulgaris* reduce a high-sucrose diet induced oxidative stress in hereditary hypertriglyceridemic rats. Pharmacol Res. 50:123–130. 15177299 10.1016/j.phrs.2003.12.013

[CIT0036] Suh WS, Lee SR, Kim CS, Moon E, Kim SY, Choi SU, Kang KS, Lee KR, Kim KH. 2016 A new monoacylglycerol from the fruiting bodies of *Gymnopilus spectabilis*. J Chem Res (S). 40:156–159.

[CIT0037] Sul D, Kim H-S, Lee D, Joo SS, Hwang KW, Park S-Y. 2009 Protective effect of caffeic acid against beta-amyloid-induced neurotoxicity by the inhibition of calcium influx and tau phosphorylation. Life Sci. 84:257–262. 19101570 10.1016/j.lfs.2008.12.001

[CIT0038] Wang Q, Sun Y, Yang B, Wang Z, Liu Y, Cao Q, Sun X, Kuang H. 2014 Optimization of polysaccharides extraction from seeds of *Pharbitis nil* and its anti-oxidant activity. Carbohydr Polym. 102:460–466. 24507306 10.1016/j.carbpol.2013.11.068

[CIT0039] Wang Z-M, Cai P, Liu Q-H, Xu D-Q, Yang X-L, Wu J-J, Kong L-Y, Wang X-B. 2016 Rational modification of donepezil as multifunctional acetylcholinesterase inhibitors for the treatment of Alzheimer’s disease. Eur J Med Chem. 123:282–297. 27484514 10.1016/j.ejmech.2016.07.052

[CIT0040] Wettasinghe M, Shahidi F, Amarowicz R. 2002 Identification and quantification of low molecular weight phenolic antioxidants in seeds of evening primrose (*Oenothera biennis* L.). J Agric Food Chem. 50:1267–1271. 11853516 10.1021/jf010526i

[CIT0041] Wilcock GK, Lilienfeld S, Gaens E. 2000 Efficacy and safety of galantamine in patients with mild to moderate Alzheimer’s disease: multicentre randomized controlled trial. BMJ. 321:1–7. 11110737 10.1136/bmj.321.7274.1445 PMC27547

[CIT0042] Wu L, Tong T, Wan S, Yan T, Ren F, Bi K, Jia Y. 2017 Protective effects of puerarin against Aβ 1-42-Induced Learning and Memory Impairments in Mice . Planta Med. 83:224–231. 27420352 10.1055/s-0042-111521

[CIT0043] Xu T, Shen X, Yu H, Sun L, Lin W, Zhang C. 2016 Water-soluble ginseng oligosaccharides protect against scopolamine-induced cognitive impairment by functioning as an antineuroinflammatory agent. J Ginseng Res. 40:211–219. 27635118 10.1016/j.jgr.2015.07.007 PMC5005308

[CIT0044] Yehuda S, Rabinovtz S, Carasso RL, Mostofsky DI. 1996 Essential fatty acids preparation (Sr-3) improves Alzheimer’s patients quality of life. Int J Neurosci. 87:141–149. 9003975 10.3109/00207459609070833

[CIT0045] Zee OP. 2009 Pharmacognosy. Seoul, Korea: Sunkyunkwan University Press.

[CIT0046] Zhao Y, Dou J, Wu T, Aisa HA. 2013 Investigating the antioxidant and acetylcholinesterase inhibition activities of *Gossypium herbaceam*. Molecules. 18:951–962. 23344203 10.3390/molecules18010951 PMC6269909

